# Parallelized microscale fed-batch cultivation in online-monitored microtiter plates: implications of media composition and feed strategies for process design and performance

**DOI:** 10.1007/s10295-019-02243-w

**Published:** 2019-10-31

**Authors:** Holger Morschett, Roman Jansen, Christian Neuendorf, Matthias Moch, Wolfgang Wiechert, Marco Oldiges

**Affiliations:** 1 Institute of Bio- and Geosciences, IBG-1: Biotechnology, Forschungszentrum Jülich GmbH, Jülich, Germany; 2 Computational Systems Biotechnology, RWTH Aachen University, Aachen, Germany; 3 Institute of Biotechnology, RWTH Aachen University, Aachen, Germany

**Keywords:** *Corynebacterium glutamicum*, Protocatechuic acid, Fed-batch, Microfluidic microtiter plate, Protein secretion

## Abstract

Limited throughput represents a substantial drawback during bioprocess development. In recent years, several commercial microbioreactor systems have emerged featuring parallelized experimentation with optical monitoring. However, many devices remain limited to batch mode and do not represent the fed-batch strategy typically applied on an industrial scale. A workflow for 32-fold parallelized microscale cultivation of protein secreting *Corynebacterium glutamicum* in microtiter plates incorporating online monitoring, pH control and feeding was developed and validated. Critical interference of the essential media component protocatechuic acid with pH measurement was revealed, but was effectively resolved by 80% concentration reduction without affecting biological performance. Microfluidic pH control and feeding (pulsed, constant and exponential) were successfully implemented: Whereas pH control improved performance only slightly, feeding revealed a much higher optimization potential. Exponential feeding with *µ* = 0.1 h^−1^ resulted in the highest product titers. In contrast, other performance indicators such as biomass-specific or volumetric productivity resulted in different optimal feeding regimes.

**Electronic supplementary material:**

The online version of this article (10.1007/s10295-019-02243-w) contains supplementary material, which is available to authorized users.

## Background

To achieve economic goals such as improved yield and productivity, reduction of substrate excess inhibition, limitation of by-product formation, oxygen demand and coupled heat generation, the majority of industrial bioprocesses are operated in fed-batch mode [4, 19]. Otherwise, the development of bioprocesses typically necessitates extensive screening of strain candidates and process parameters. Due to limited experimental capacities and extensive manual workload, such investigations cannot be performed efficiently in laboratory stirred-tank reactors (STRs). Instead, small-scale tools such as shake flasks and microtiter plates (MTPs) have long been the tools of choice for such tasks. A comprehensive discussion of these cultivation systems and systematic strategies for scale-up can be found elsewhere [12, 20, 21, 27]. Due to technical restrictions, many of the available systems remain limited to batch cultivation; therefore, batch mode screening is a frequently used standard strategy. However, the transferability of results acquired in small-scale batch experiments to fed-batch environments has to be evaluated critically [13, 35].

Addressing this critical issue, a variety of concepts enabling feeding in screening-scale devices has been developed and some of the listed technologies are available as commercial products. Table 1 compares selected technological concepts focusing on specific features and limitations.

**Table 1:** Comparison of selected feeding technologies for mini- and microbioreactor devices

Technology	Realized in			Key features	Limitations
	Shake flask	MTP	Micro-STR		
Diffusive release from polymer matrix	[13, 34]^a^	[34, 39]^a^	–	Ease of use	Relatively slow rates, constant feed only
Diffusive release from reservoir via membrane	[1, 31, 32]	–	–	Good flexibility	Constant feed only, high characterization effort
Enzymatic digestion of non-metabolizable polymer	[17]	[6, 16, 17, 30, 36]	–	Ease of use	Rate control difficult, narrow operating window
Dosing via liquid handling robot	–	[10]^a^	[33]^a^	High flexibility	Intermittent feed only, high invest
Dosing via pumps	[14]^a^	[2, 8]^a^	[9]	High flexibility	High complexity, investment and operating costs

^a^Integrated system solution commercialized

While most diffusive and enzymatic systems enable fast implementation and simple use, they typically involve a set of significant drawbacks. Diffusive release of nutrients either from a homogeneous insoluble polymer or via a membrane is driven by the concentration gradient between the reservoir and culture suspension. Such approaches are, therefore, limited to constant feeding under typical operating conditions and release rates in the lower range. Systems relying on enzymatic digestion of an inert polymer into metabolizable monomers suffer from insufficiently characterized enzymes, susceptible to pH change during cultivation as well as distinct medium components often displaying a dramatic influence on activity and stability. Moreover, microorganisms showing an intrinsic capability for degradation of polymer substrate, such as many species of fungi, cannot be utilized by such concepts. Stable operation with tightly controlled release rates is thus only possible within rather narrow operating windows and not at all for a wide range of relevant production hosts.

In contrast, pumps and robot-assisted dosing systems are not affected by such implications, but implementation into laboratory infrastructure as well as adequate operation may be challenging. Moreover, such sophisticated technologies typically involve significant investments and operating costs. If flexible operability, strict control, complex and/or feedback-regulated feeding profiles are mandatory, such devices are the preferred approach. For further details and discussion of selected feeding and nutrient release technologies for small-scale cultivation systems, see [17, 21].

In the context of these emerging technologies, a protocol for microscale cultivation of the GFP-secreting *Corynebacterium glutamicum* was developed and validated. Since GFP can easily be monitored online in a non-invasive manner, this strain reflects a model system for heterologous protein secretion with *Corynebacteria*. The new workflow enables flexible fed-batch operation in MTPs with active pH control and online process monitoring. Special emphasis was placed on the influence of adequate medium composition enabling reliable culture monitoring by optical methods as well as reflecting the nutritional needs of the host metabolism.

## Results and discussion

The BioLector Pro microbioreactor hardware used in this study relies on specialized microfluidic MTPs. Out of a total of 48 wells, 32 wells are intended for cultivation purposes allowing online measurement of biomass via scattered light, fluorescence and finally pH and dissolved oxygen (DO) via immobilized dyes (optodes). For each set of four cultivation wells, two reservoir wells are available from which liquid can be pumped via feedlines into their corresponding cultivation wells. A detailed description of the hardware and underlying principles is given elsewhere [2, 8]. Realizing fed-batch operation in such MTPs requires careful adaptation of operating procedures to meet demands such as accurate pH measurement for reliable pH titration as well as suitable design of cultivation media and feed solutions with respect to biological requirements.

### Adaptation of culture medium allowing accurate optical pH measurement

#### Protocatechuic acid interferes with pH measurement

Reliable pH control requires the accurate measurement of actual process values. In the given case of optical measurement using an immobilized pH optode (ex/em = 470/520 nm), there is a risk of interferences by medium components being optically active in the range of measured wavelengths [18]. Preliminary experiments (data not shown) indicated that this holds true for protocatechuic acid (PCA), which is present in the applied CGXII medium (see Sect. 4.1). Thus, the first step addressed the investigation of potential interference of PCA with optical pH measurement. Therefore, CGXII media with different PCA concentrations were incubated under cultivation conditions but without applying any biological system.

**Fig. 1:**
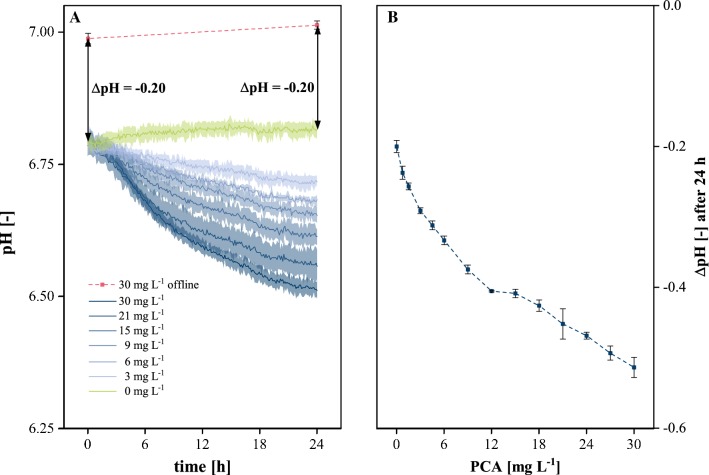
Influence of PCA concentration on optical pH measurement under typical cultivation conditions. **a** Time-resolved influence on optical measurement for selected concentrations in comparison to offline measurement with standard electrochemical pH probe. **b** Difference in optical online pH measurements at different PCA concentrations compared to PCA-free medium after 24 h incubation time; 30 °C, 1300 rpm, *V*_L_ = 800 µL, relative humidity ≥ 85%. Confidence tubes and error bars deviated from technical replicates (*n* ≥ 3)

As monitored by electrochemical offline measurement, the pH increased only marginally during 24 h incubation of cell-free standard CGXII (CGXIIstd) containing 30 mg L^−1^ PCA. Similar behavior was observed with optical measurements of PCA-free medium with a consistent offset of ∆pH = − 0.20 observed between the two methods. Applying different PCA concentrations, the initial offset remained fairly constant but deviation steadily increased with incubation time in the case of higher PCA concentrations correlated with a higher deviation over time (Fig. 1a, b). As depicted in Fig. 1b, the offset between standard electrochemical and optical pH measurement after an incubation time of 24 h developed up to ∆pH = − 0.52 for 30 mg L^−1^ PCA.

Thus, the deviation in the optical measurements could be assigned to PCA interfering with the pH optode material rather than to actual pH changes of the medium. Regarding pH regulation driven by optical measurements, a constant offset can easily be compensated by appropriately adjusting the regulation setpoint or a calibration offset whereas creeping changes over time could result in pH control to a wrong value. As PCA represents an essential component for reproducible growth of *C.* *glutamicum* in CGXII media [38] and the alternatives described in the literature [22] were not feasible in this context due to price, potential carcinogenicity or significant contribution to the carbon source pool of the medium, possible reduction of PCA concentration was addressed to keep measurement interference within an acceptable range.

**Fig. 2:**
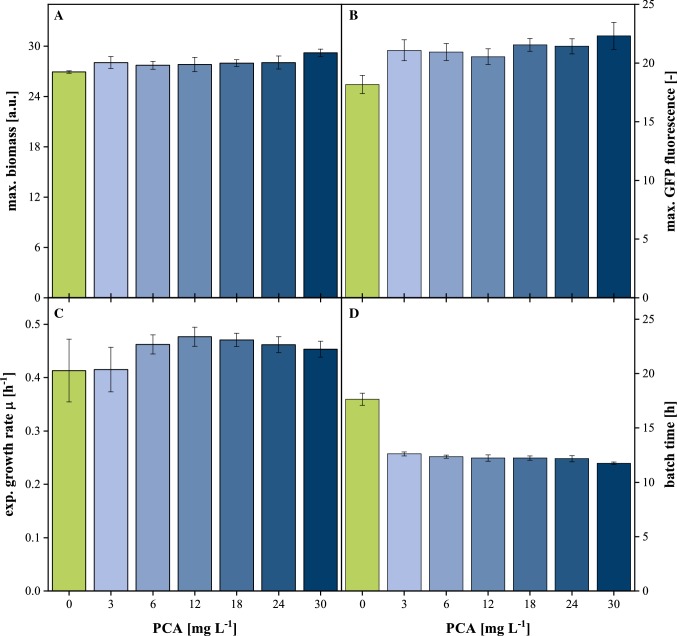
Performance indicators of *C.* *glutamicum* batch cultivation with varying PCA concentration. **a** Maximum biomass by scattered light, **b** maximum GFP fluorescence, **c** growth rate during exponential phase, **d** time until depletion of carbon source by means of DO signal; 20 g L^**−**1^ glucose, 30 °C, 1300 rpm, *V*_L_ = 800 µL, relative humidity ≥ 85%. Error bars deviated from biological replicates (*n* ≥ 4)

Therefore, a series of cultivation experiments was conducted targeting the influence of reduced PCA concentration on the growth phenotype of *C.* *glutamicum*. While Fig. 2 provides an overview by means of performance indicators, the underlying growth profiles are presented in Supplementary Figure S1.

As depicted in Fig. 2a, the reduction of PCA concentration to 3 mg L^−1^ did not result in observable changes in achieved biomass and only complete omission of the compound reduced biomass concentration by approx. 5%. A similar pattern was observed for the detected GFP fluorescence. Here, measurements could not be distinguished on the basis of standard error between biological replicates (± 3.7%) and only 0 mg L^−1^ PCA resulted in 18.6% loss of GFP fluorescence signal (Fig. 2b). A different pattern was revealed with respect to exponential growth rate (Fig. 2c) where a reduction to 6 mg L^−1^ PCA had no observable impact. Concentration lower than 6 mg L^−1^ resulted in slightly reduced growth rates, but more importantly in increasing fluctuation between biological replicates with the standard deviation rising from 3.3% for cultivation with 6 mg L^−1^ to 14.2% for 0 mg L^−1^ PCA. In parallel, the reduction of PCA concentration led to an incremental increase of batch time as detected by the sharp rise of DO upon depletion of the primary carbon source. As a consequence, batch time was prolonged by 7.3% at 3 mg L^−1^ and even increased by 50% if PCA was completely omitted from the medium (Fig. 2d).

In summary, the reduction of PCA concentration mainly affected growth rate and batch time, but as a general principle, lowering the concentration below critical thresholds typically resulted in significantly reduced reproducibility between biological replicates. This observation is supported by current reports from Lindemann et al. whose microfluidic cultivation experiments with *C.* *glutamicum* growing on PCA as sole carbon source revealed, besides other effects, significant influence of PCA concentration on doubling time and cell-to-cell variability [25]. Anyway, for the given process, batch time proved to be the most sensitive parameter with respect to PCA variation. From this perspective, complete elimination of PCA from the medium was not possible, but a reduction to 6 mg L^−1^ represented an acceptable compromise with respect to biological performance. It should be noted that applying conditions without additional plasmid induction by IPTG may allow further reduction of PCA concentration. For example, non-induced conditions enabled a reduction to 3 mg L^−1^ without any observable phenotype on the same strain (Supplementary Figure S2).

Furthermore, earlier studies revealed that *C.* *glutamicum* co-metabolizes PCA alongside glucose. Typical times up to depletion depend on initial cell density and are in the range of a few hours [38], but as yet there is still no exact quantitative evaluation of PCA uptake. As it could be expected that interference of PCA with optical pH measurement will no longer occur as soon as PCA has been consumed by the microorganisms, cell-free CGXII media with selected PCA concentrations were incubated under cultivation conditions and the pH was monitored by optical measurements. After 5 h incubation, all media were removed, the MTP wells washed and refilled with PCA-free medium. For better visibility of deviation, time series are shown after subtracting the respective optical measurement in PCA-free medium.

**Fig. 3:**
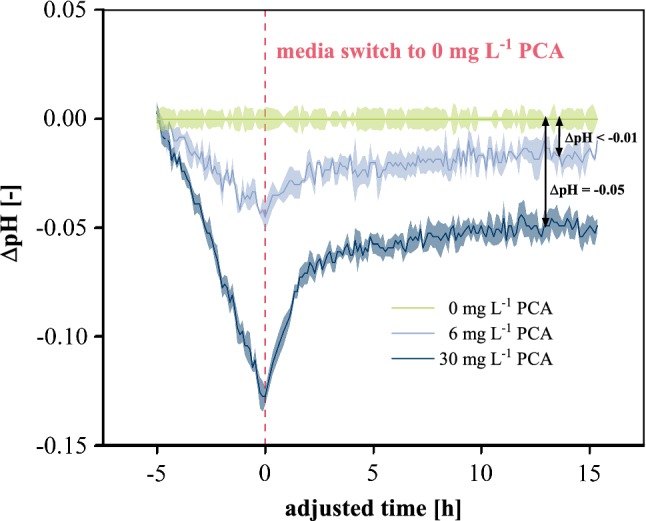
Investigation of PCA interference. After 5 h of incubating cell-free CGXII media with selected PCA concentrations, all media were replaced with PCA-free CGXII. All measurements were corrected by the value of the corresponding measurement in PCA-free medium; 30 °C, 1300 rpm, *V*_L_ = 800 µL, relative humidity ≥ 85%. Confidence tubes deviated from technical replicates (*n* = 3)

As can be seen in Fig. 3, 5 h incubation at standard concentration led to a measurement error of ∆pH = − 0.13 while 6 mg L^−1^ PCA caused a mismatch of < −0.05 which is in good agreement with the previous results (Fig. 1). It can be seen that replacement with PCA-free media after 5 h of incubation time led to partial regeneration of the pH optodes. Within 2.5 h, the mismatch was reduced by 48 and 58% and after an additional 10 h incubation, the absolute mismatches were ∆pH < −0.01 and ∆pH = −0.05 for 6 and 30 mg L^−1^ initial PCA concentration, respectively. Thus, it can be stated that reducing PCA to 6 mg L^−1^ yields almost complete regeneration of measurement capability, if PCA is consumed within 5 h post-inoculation while the presented data indicate that applying standard 30 mg L^−1^ PCA causes stronger and potentially permanent impairment to the optodes. It could be speculated that the aromatic character of PCA allows adsorption to or intercalation with the polymer support matrix of the optode material leading to the observed partly irreversible effects.

In the final step, the newly fixed operating point at 6 mg L^−1^ was validated in an additional cultivation experiment. To this end, biological replicates were grown with 6 mg L^−1^ initial PCA concentration and optical online pH measurement was complemented by electrochemical offline acquisition from sacrifice wells. Online pH was recalibrated to the initial medium pH measured by the electrochemical method.

**Fig. 4:**
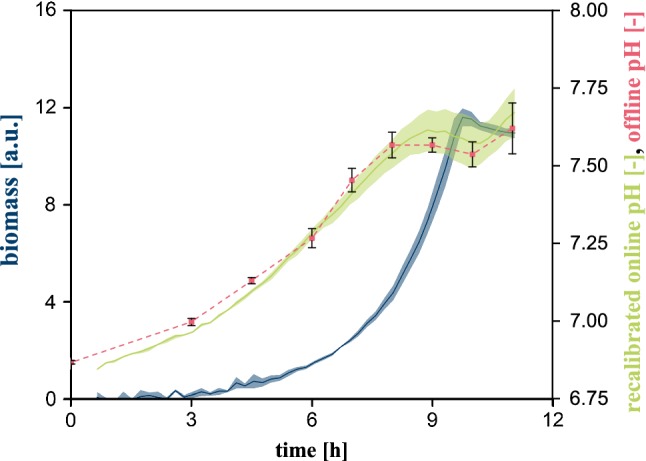
Comparison of online and offline pH during cultivation with 6 mg L^**−**1^ initial PCA concentration; 10 g L^**−**1^ glucose, 30 °C, 1300 rpm, *V*_L_ = 800 µL, relative humidity ≥ 85%. Confidence tubes and error bars deviated from biological replicates (*n* ≥ 3)

As shown in Fig. 4, the online and offline data are in good agreement throughout the complete cultivation experiment of *C.* *glutamicum*. Along all the offline samples, the observed mismatch between the two orthogonal methods was never higher than ∆pH = 0.05. Consequently, the current setup offers the option of reliable and accurate online pH measurement via the integrated optodes, which is the essential prerequisite for appropriate pH control on the microscale. This is facilitated by reducing the applied PCA to 20% of its standard concentration, which does not affect biological performance.

#### Influence of nitrogen source

As discussed earlier in this section, each cultivation well is connected to two reservoir wells for microfluidic supply. In the case of fed-batch operation, one of these lines is needed to supply the feed solution and, therefore, only one remains for one pH-controlling agent. Thus, in contrast to laboratory-scale stirred-tank reactors, pH control is limited to single-sided titration. This has direct implications for media design since it needs to be ensured that single-sided pH control is sufficient.

CGXIIstd features two nitrogen sources, i.e., ammonium sulfate and urea. While the direct application of ammonium with the incorporation of NH_3_ to the biomass leads to the acidification of the cultivation medium by accumulation of the remaining H^+^ [5], the pattern is different for utilization of urea. Urea cannot directly be incorporated by the cells but is first hydrolyzed by an intracellular urease releasing two ammonium and one carbonate. While ammonium obtained in this way is used by the cells in the same way as its mineral equivalent deviating from ammonium sulfate, the corresponding pH-lowering effect is compensated. Under typical cultivation conditions, neutral pH values force the carbonate equilibrium towards HCO_3_^−^, removing free H^+^ from the medium and thereby increasing the pH. As long as the rate of H^+^ binding by CO_3_^2−^ is higher than the corresponding release rate by incorporation of NH_3_ into the biomass, a net pH increase occurs. In contrast, if the ratio of both rates changes in the opposite direction, the pH drops.

A combination of both nitrogen sources present in CGXIIstd may, therefore, lead to complex pH patterns that cannot be controlled by single-sided pH control and reduction to a single nitrogen source is preferred. Although using ammonium sulfate as sole nitrogen source combined with an alkaline reagent for pH control seems to be the easier and generally applicable way at first sight, for this specific case, a different strategy is chosen. A previous study with the same strain revealed that urea is the superior nitrogen source for efficient secretory protein production (30% increase in protein titers by omitting ammonium sulfate from CGXII media) [7]. This product titer increase was considered a major advantage and urea as sole nitrogen source was preferred. Targeting the fed-batch mode requires nutritional balancing and, therefore, urea concentration profiling was performed in the next step to determine the amount of urea needed to convert a given amount of carbon source (10 g L^−1^ glucose) into biomass.

**Fig. 5:**
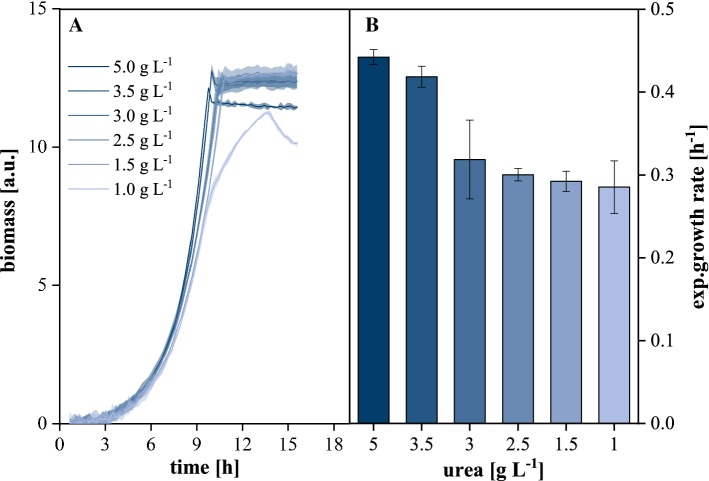
Urea concentration profiling during batch growth of *C.* *glutamicum*. **a** Growth curves by scattered light, **b** Growth rates during the exponential phase; 10 g L^**−**1^ glucose, 30 °C, 1300 rpm, *V*_L_ = 800 µL, relative humidity ≥ 85%. Confidence tubes and error bars deviated from biological replicates (*n* ≥ 3)

As shown in Fig. 5a, a reduction of urea concentration to 1.5 gL^−1^ did not result in reduced biomass concentrations and was thus sufficient for conversion of 10 g L^−1^ glucose from a stoichiometric point of view while 1 g L^−1^ resulted in a 7.3% reduction of achievable biomass. However, such reductions had remarkable effects on growth kinetics as any tested reduction resulted in a significant decrease of the exponential growth rate (Fig. 5b) while the lowest concentration even resulted in a slow shift from exponential towards linear kinetics within the last 4 h before glucose depletion (9.75–13.65 h). It seemed that beyond stoichiometric nitrogen requirements, there might be a kinetic limitation by either urea uptake or hydrolysis at reduced extracellular urea availability and consequently, urea had to be supplied in sufficient quantities to avoid such limitations during glucose feeding. For upcoming fed-batch experiments, this was ensured by a 4:1 (w w^−1^) co-feed of glucose and urea (Sect. 4.3).

### Evaluation of different process strategies

**Fig. 6:**
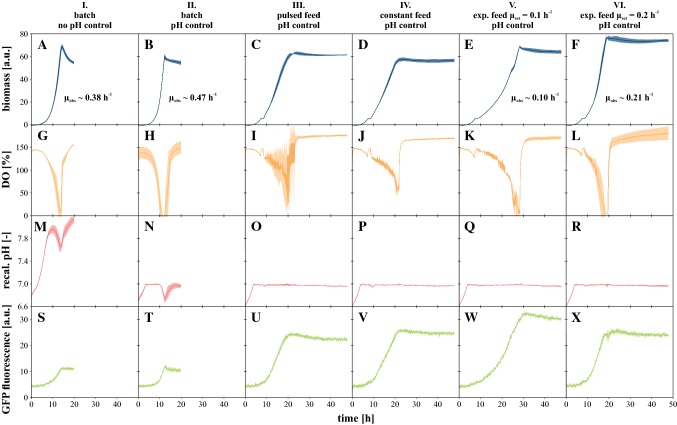
Comparison of different process strategies from parallelized microfluidic cultivation ordered in columns: batch without (I) and with pH control (II), as well as pulsed (III), constant (IV) and exponential feeding with pH control (V, VI). **a**–**f** Biomass by scattered light, **g**–**l** DO, **m**–**r** recalibrated pH, **s**–**x** GFP fluorescence; 40 g L^**−**1^ glucose, 30 °C, 1400 rpm, *V*_init_ = 800 µL, titration to pH 7 with 3 M H_3_PO_4_, relative humidity ≥ 85%. Error bars deviated from biological replicates (*n* ≥ 3)

Having addressed the necessary adaptations of the CGXII culture medium to ensure appropriate pH measurement and control as well as having revealed the C:N nutritional balance needed for maximum growth potential, a set of different feeding profiles (pulsed, constant, exponential) were realized in pH-controlled microfluidic fed-batches. After an initial 8.5 h batch phase on 5 g L^−1^ glucose, feeding was started in the individual wells of the MTP. In all cases, a total of 70 µL was fed resulting in a total concentration of 40 g L^−1^ glucose per culture. As a reference, additional batch cultivations with and without pH control were performed providing the same amount of glucose and urea as in fed-batch cultures right from the beginning (Fig. 6). The resulting processes where compared based on the acquired online data where biomass comparison (“achieved” biomass) was always done based on the first value after detection of substrate depletion. Thereby, distortion due to potential biomass signal decrease in stationary phase could be excluded.

In the reference batch cultivation without pH control, glucose was consumed within 13.9 h and steady exponential growth was observed at *µ* ~ 0.38 h^−1^ (A) while DO correspondingly decreased to 0% at the end of the growth phase (G). Meanwhile, the pH first increased to 8.0 and subsequently dropped to 7.5 until glucose depletion (M). A growth-coupled increase of GFP fluorescence was observed up to a maximum of 11.0 ± 0.9 a.u. (S).

When the same batch experiment was performed, but with additional implementation of pH control with phosphoric acid, glucose was depleted slightly faster (12.4 h) corresponding to an increased growth rate of *µ* ~ 0.47 h^−1^. The amount of biomass obtained was reduced by 6.8% (B). The oxygen limitation previously observed was pronounced and lasted for approx. 3 h (H), which could be an explanation for the reduced biomass yield. The pH was maintained at the desired setpoint (7.0) for most of the cultivation time but temporarily decreased during O_2_ limitation (N). In the literature, it is reported that *C.* *glutamicum* produces lactate under such conditions [23] and thus acidifies the culture medium which cannot be counteracted with the implemented single-sided pH control. Maintaining a pH near the physiological optimum might explain the faster growth compared to non-controlled conditions. In parallel to slightly reduced biomass, product formation increased by approx. 14% (T).

In the series of fed-batch experiments, pulsed feeding resulted in a 4% decrease of biomass formation and substrate feeding was completed after 21.4 h (C). During such experiments, a substrate bolus of 1 µL feed solution (equals 0.4 mg glucose) was added each time the DO signal exceeded 130%, which resulted in a strongly oscillating DO during the feed phase (I). The cells were thus exposed to fluctuations in substrate availability. Especially at higher biomass concentrations, this led to DO oscillations down to limiting conditions. Despite fluctuating DO, it was possible to strictly control pH along the whole process (O). These transient phases of O_2_ limitation did not appear to be sufficient to induce the production of lactate which would significantly acidify the culture medium as in the case of batch cultivation. However, transient lactate formation under oxygen-limited conditions followed by lactate utilization in the aerobic phase most likely occurs, as has been shown in scale-down bioreactor experiments with *C.* *glutamicum* [24]. Overall, this feeding strategy increased product formation by a factor of 2.27× (U) compared to the batch reference (S).

Analogous to the pulsed strategy, continuous rate feeding (5.22 µL h^−1^; equals 2.09 mg h^−1^ glucose) yielded a steady increase in biomass. In total, the final biomass concentration is reduced by 11% compared to the reference and the time to glucose depletion was 21.9 h (D). At the chosen feed rate, DO never dropped below 55% so that a limitation could be completely excluded (J) while the pH was successfully maintained at the desired setpoint throughout the whole process (P). A further increase in GFP formation up to 2.36-fold the reference was observed (V).

To compensate for changing growth rates as typically occurring in the feeding scenarios described above, exponential feeding is the method of choice to maintain a metabolic steady state during fed-batch culture. In this study, *µ*_set_ = 0.1 h^−1^ was applied and a biomass concentration in the range of the reference (+ 4%) was achieved within 38.4 h process time. The measured growth rate *µ*_observed_ = 0.10 h^−1^ was in perfect agreement with *µ*_set_ even though a slight oxygen limitation occurred at the end. Starting from the (suboptimal) reference setting, i.e., batch without pH control, a slight increase in product formation (+ 14%) was achieved by implementing pH control. However, faster biomass formation increased oxygen demand and thus led to pronounced O_2_ limitation, which might have limited the increase in product formation. Controlled substrate supply had a much stronger positive influence on GFP production. For the cases tested, average improvement was ~ 140% and optimal production (+ 175%) was achieved by exponential feed at *µ*_set_ = 0.1 h^−1^. These observations are in good agreement with the literature, where limiting microbial growth has repeatedly been reported to be coupled with increased product formation [3, 11, 29, 37]. Although the exponential feed at low *µ*_set_ was optimal with respect to the product titer achieved, other performance indicators are frequently important as well. Among others, they include biomass-specific product formation and volumetric productivity.

**Fig. 7:**
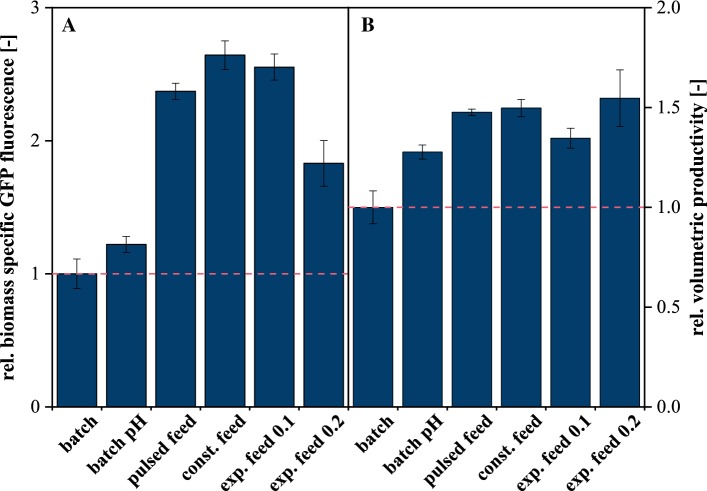
Influence of different process strategies on key performance indicators. **a** Biomass-specific GFP fluorescence, **b** volumetric productivity; 40 g L^**−**1^ glucose, 30 °C, 1400 rpm, *V*_init_ = 800 µL, titration to pH 7.0 with 3 M H_3_PO_4_, relative humidity ≥ 85%. Error bars deviated from biological replicates (*n* ≥ 3)

Biomass-specific product formation is a key factor to describe the efficiency of cellular product formation. As depicted in Fig. 7a, pH control during batch cultivation slightly improves biomass-specific GFP fluorescence (+ 22%), most probably due to a reduced energy metabolite need to maintain intracellular pH at the physiological optimum. This comparatively low increase in product formation may be attributable to the nature of the product and the synthesis itself. It can be assumed that IPTG-induced GFP production is only marginally influenced by pH as long as the latter remains within the physiological operation window, this will most likely be different for other product families such as organic acids [26].

A much more pronounced improvement was achieved by limiting carbon supply during feeding experiments. Here, protein production was up to 2.37× reference (*µ*_set_ = 0.1 h^−1^), but constant and also pulsed feeding provided a similar performance while increased carbon supply (*µ*_set_ = 0.2 h^−1^) reduced efficiency significantly (1.83× reference). From this perspective, slow exponential feeding represents the optimal process strategy. It is striking that the performance of pulsed feeding was comparable to constant and slow exponential feed profiles although such a setup results in rapidly fluctuating nutrient supply rather than steady carbon limitation. This is most likely attributable to the nature of the expression cassette where IPTG was used to induce strictly growth-coupled GFP production. For systems with more complex regulation patterns, such as substrate excess inhibition, it can be assumed that pulsed feeding will be outperformed by profiles resulting in steadily limiting conditions.

In industrial production, timescales are of significance in addition to metabolic efficiency, resulting in a different picture (Fig. 7b). With respect to volumetric productivity, pulsed, constant and exponential feeding at *µ*_set_ = 0.2 h^−1^ provided the best performance (~ 1.5× reference). Although exponential feeding at *µ*_set_ = 0.1 h^−1^ was more efficient in terms of metabolism, the prolonged feed phase (almost 10 h longer) resulted in significant performance loss (1.35× reference). Interestingly, this was also on a level comparable to pH-controlled batch cultivation.

From this application study, it seems that for the given production system simple feeding strategies such as constant rate nutrient supply can compete or even outperform elaborate profiles such as exponential feeding while simultaneously requiring less process knowledge and instrumentation for large-scale realization. This might be an argument for frequently preferring simple feeding profiles for industrial-scale process realization, but nevertheless, overgeneralizations should be avoided as optimal feeding regimes might easily differ for production systems other than the one used in this study.

## Conclusions

A workflow was developed for pH-controlled fed-batch cultivation of a protein-producing *Corynebacterium glutamicum* strain at microliter scale. Relying on microfluidic microtiter plates, up to 32 parallel cultures were run including online monitoring of biomass concentration (scattered light), dissolved oxygen, pH and biogenic fluorescence(s).

In the first step, interference of protocatechuic acid, an essential component for reproducible growth of *Corynebacterium* in defined CGXII medium, was revealed with optical pH measurement, which is likely due to adsorption to or intercalation of PCA with the optode matrix polymer. The commonly used 30 mg L^−1^ resulted in significant and only partially reversible measuring offsets of > 0.5 pH within 24 h of incubation compared to standard electrochemical methods. This clearly represents a general drawback of optical measurements techniques used in many high-throughput devices and thus, validation against orthogonal methods seems highly recommendable if not only qualitative trends but also absolute values are needed. Systematic evaluation enabled the PCA concentration to be reduced to 6 mg L^−1^ while maintaining growth and protein production phenotype. Even lower concentrations resulted in slower growth, prolonged lag phase duration and reduced overall process reproducibility. Applying 6 mg L^−1^, a very good agreement between electrochemical offline and optical online pH measurement was achieved, representing the necessary basis for microfluidic pH control according to optical signals.

The media composition was then adjusted so that pH could be controlled by single-sided titration with phosphoric acid: The applied CGXII medium features two nitrogen sources, namely ammonium sulfate and urea potentially leading to complex pH patterns that cannot be properly controlled by single-sided pH control. In general, use of ammonium sulfate as the sole nitrogen source combined with an alkaline agent might seem preferable as it facilitates pH control for processes or process phases featuring media acidification. Nevertheless, according to the literature, ammonium sulfate is disadvantageous with respect to protein production using *C.* *glutamicum* and it was, therefore, omitted from the medium. Consequently, urea was used as the sole nitrogen source. Furthermore, it was shown that urea concentrations far beyond stoichiometric needs are mandatory to ensure that the growth performance of *Corynebacterium* is not affected thus indicating an as-yet-unrevealed kinetic limitation, either urea uptake or hydrolysis at low urea concentrations.

Having adapted the defined culture medium, a series of microfluidic fed-batch processes was conducted while the pH was strictly controlled within the optimal physiological window. An additional reference batch under pH-controlled conditions revealed that, compared to buffered batch processes, production performance profited from keeping pH stable at the physiological optimum. The greatest improvement was achieved by limiting carbon supply by feeding, an increase in product concentration of up to 175% was achieved by exponential feeding at *µ*_set_ = 0.1 h^−1^. Considering other performance indicators such as volumetric productivity revealed the advantage of simple feeding strategies over more complex approaches, due to shorter cultivation time and reduced complexity representing a significant advantages for potential scale-up to laboratory or pilot scale. Nevertheless, such a setup clearly offers great potential to significantly increase time efficiency during early bioprocess development and strain phenotyping where numerous biological and engineering design parameters need to be addressed during parallel experimentation to identify ideal values. Additionally, changing from batch operation to pH-controlled fed-batch represents a substantial step towards conducting early upstream development under conditions relevant at an industrial scale which is particularly relevant during screening of producer strain libraries.

## Methods

### Chemicals, strain and media

All chemicals were obtained from Sigma-Aldrich (Steinheim, Germany) or Roth (Karlsruhe, Germany) and were of analytical grade.

*Corynebacterium glutamicum* ATCC13032 was used as the model strain for this study. For all cultivation experiments addressing the establishment of the method, a variant carrying the pEKEx2 plasmid for IPTG-inducible expression of a pCGPhoD^Cg^–GFP fusion protein secreted via the twin-arginine translocation pathway [28] was used while kanamycin served as the selection marker. The strain was kindly provided by Prof. Dr. Roland Freudl (Forschungszentrum Jülich GmbH).

All cultivations were performed in defined CGXII medium [15]. While individual variants are described in “Results and discussion”, the original medium CGXIIstd had the following initial composition: 41.85 g 3-(*N*-morpholino)propanesulfonic acid, 10 or 20 g glucose, 20 g (NH_4_)_2_SO_4_, 5 g (NH_2_)_2_CO, 1 g KH_2_PO_4_, 1 g K_2_HPO_4_, 250 mg MgSO_4_·7H_2_O, 13.25 mg CaCl_2_·2H_2_O, 50 mg kanamycin sulfate, 30 mg protocatechuic acid, 10 mg FeSO_4_·7H_2_O, 10 mg MnSO_4_·H_2_O, 1 mg ZnSO_4_·7H_2_O, 0.313 mg CuSO_4_·5H_2_O, 0.2 mg biotin, and 0.02 mg NiCl_2_·6H_2_O. Using 4 M NaOH, the pH value was set to 7. If necessary, 59.58 mg L^−1^ (250 mmol L^−1^) isopropyl-*ß*-d-thiogalactopyranoside was added to induce protein expression.

### Strain maintenance and pre-cultivation

Cells were harvested during exponential growth, centrifuged for 5 min at 9283*g* in a GS-15R (Beckman Coulter, Krefeld, Germany), resuspended to OD 40 in 0.9% (w v^−1^) NaCl containing 25% (w v^− 1^) glycerol, and 1 mL aliquots were frozen to − 80 °C for storage.

For pre-cultivation, 50 mL CGXIIstd medium was incubated in a fourfold baffled 500 mL shake flask at 30 °C, 300 rpm and 25 mm shaking diameter in a Multitron Standard shaking incubator (Infors, Einsbach, Germany). Depending on the experiment timing, the inoculum from cryocultures was varied in such a way that at harvest sufficient concentrations of biomass were available without exceeding the maximum oxygen transfer capacity of the shake flask system (according to preliminary experiments, this was reached when an optical density of 20 was exceeded). The culture was harvested, centrifuged for 5 min at 9283*g* in a GS-15R and resuspended in 0.9% (w v^−1^) NaCl to obtain a stock suspension for the subsequent main cultures.

### Microbioreactor cultivation

All main cultivations were run in microtiter plates using a BioLector Pro microbioreactor system (m2p-labs, Baesweiler/Germany) at 30 °C, either 1300 or 1400 rpm shaking frequency, 35% headspace oxygen concentration and ≥ 85% relative humidity. For batch experiments without pH control, MTP-48-BOH1 FlowerPlates covered by F-GP-10 gas-permeable sealing foils were used while microfluidic experiments were conducted in MTP-MF32-BOH1 microfluidic FlowerPlates covered by gas-permeable sealing foils for microfluidics F-GPRSMF32-1 (all m2p-labs, Baesweiler/Germany). Biomass (scattered light), GFP fluorescence, pH and DO were measured for each cultivation well at 15 min cycle time in standard batch experiments and at 5 min cycle time in microfluidic experiments. All fed-batch experiments were conducted with a feeding solution containing 400 g L^−1^ glucose and 100 g L^−1^ urea applying the feeding profiles given in Table 2.

**Table 2:** Representation feeding strategies applied

Profile	Equation	Variables
Constant feed	$\dot{V}\left( t \right) = \dot{V}_{\text{const}}$	$\dot{V}_{\text{const}}$ constant feed term (µL h^−1^)
Exponential feed	$\dot{V}\left( t \right) = \dot{V}_{\text{init}} e^{{\mu_{\text{set}} t}}$ with $\dot{V}_{\text{init}} = \left( {\frac{{\mu_{\text{set}} }}{{Y_{{{\text{X}}/{\text{S}}}} }} + m_{\text{S}} } \right)\frac{{c_{{{\text{S}},0}} Y_{{{\text{X}}/{\text{S}}}} V_{\text{init}} }}{{c_{\text{S,F}} }}$	$\dot{V}_{\text{init}}$ initial feed rate (µL h^−1^) $\mu_{\text{set}}$ target exponential growth rate (h^−1^) $Y_{\text{X/S}}$ biomass yield (g g^−1^)^a^ $m_{\text{S}}$ maintenance coefficient (g g^−1^ h^−1^)^b^ $c_{{{\text{S}},0}}$ substrate concentration at batch start (g L^−1^) $V_{\text{init}}$ reactor volume at feed start (µL) $c_{\text{S,F}}$ substrate concentration in feed (g L^−1^)
Pulsed feed	$V_{\text{pulse}}$^c^	$V_{\text{pulse}}$ volume per pulse (µL)

^a^0.61 g g^−1^ from previous experiments

^b^0.02 g g^−1^ h^−1^ from previous experiments

^c^Single pulse dosed each time DO exceeded 130%

Relying on optical pH measurement, from 1 h post-inoculation single-sided regulation to pH 7 was used in microfluidic experiments. In all cases 3 M H_3_PO_4_ was used as the titration agent and controller settings were *P* = 1.0, *I* = 1.0 and a deadband of 0.05.

All solutions fed during microfluidic experiments were filtered with 0.2 µm cellulose acetate syringe filters (DIA-Nielsen, Düren/Germany) to prevent any blocking of microfluidic channels by potentially suspended solids.

Each time series of biomass (scattered light) was blanked by the average of its initial five measurement cycles. To compensate for dilution by feeding and pH titration, intensity measurements (scattered light and GFP fluorescence) were corrected by the quotient of the actual and initial volume of the respective culture.

### Off-line analytical methods

#### Biomass

Optical density was recorded at 600 nm against desalted water as a blank using 10-mm polystyrene semi-micro cuvettes (ratiolab, Dreieich/Germany) and a UV-1800 photometer (Shimadzu, Duisburg/Germany). Samples were appropriately diluted in 0.9% (w v^−1^) NaCl to match the linear range of the device (0.1–0.3) whenever necessary.

#### pH

Offline pH was acquired without any pretreatments using an S20 SevenEasy pH meter with a 6.0234.100 micro electrode (Metrohm, Filderstadt/Germany). Using a water bath, all samples were acclimatized to 30 °C before measurement.

### Growth rate estimation

Growth rates were estimated by the exponential fit [*y*(*t*) = *y*_0_ + *A* · e^µt^] of blanked and volume-corrected scattered light measurements (Sect. 4.3) using OriginPro 2017G (OriginLab Corporation, Northampton, USA).

## Electronic supplementary material

Below is the link to the electronic supplementary material.

Supplementary material 1 (PDF 542 kb)
