# Supplementary material for: Parallelized microscale fed-batch cultivation in online-monitored microtiter plates: implications of media composition and feed strategies for process design and performance
Source: J Ind Microbiol Biotechnol. 2019 Oct 31;47(1):35–47. doi: 10.1007/s10295-019-02243-w (PMC6971147; doi:10.1007/s10295-019-02243-w)
Supplement: Supplementary file 1 — Supplementary material 1 (PDF 542 kb) [file 10295_2019_2243_MOESM1_ESM.pdf]

Parallelized microscale fed-batch cultivation in online monitored microtiter plates: Implications of media composition and feed strategies for process design and performance

Holger Morschett[1, ǂ], Roman Jansen[1,3, ǂ], Christian Neuendorf[1], Matthias Moch[1], Wolfgang Wiechert[1,2], Marco Oldiges[1,3]

[1] Forschungszentrum Jülich GmbH, Institute of Bio- and Geosciences, IBG-1: Biotechnology, Jülich
[2] RWTH Aachen University, Computational Systems Biotechnology (AVT.CSB), Aachen
[3] RWTH Aachen University, Institute of Biotechnology, Aachen
[ǂ] equal contribution
[*] corresponding author: m.oldiges@fz-juelich.de

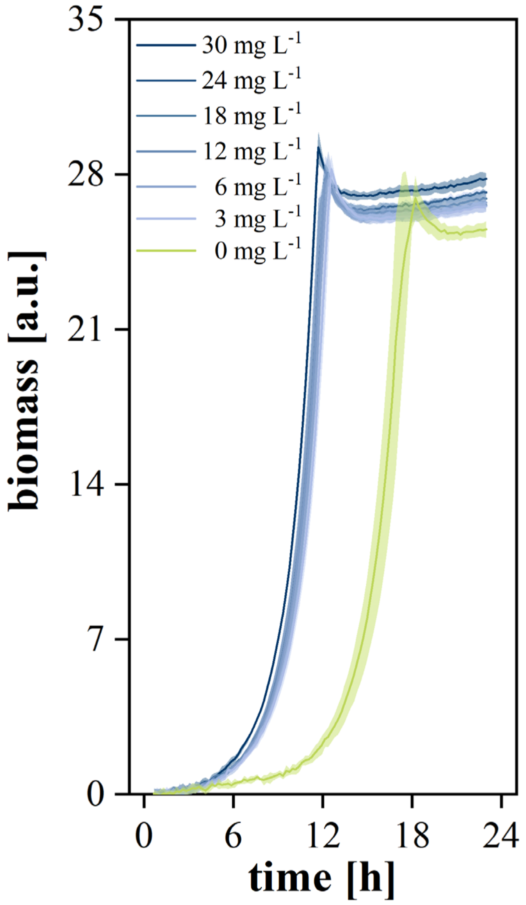

Supplementary figure S1: Batch growth of IPTG-induced *Corynebacterium glutamicum* at varying PCA concentrations; 20 g L^{-1} glucose, 0.25 mM IPTG, 30 °C, 1300 rpm, V_L = 800 µL, relative humidity ≥ 85 %. Error bars deviated from biological replicates (n ≥ 4).

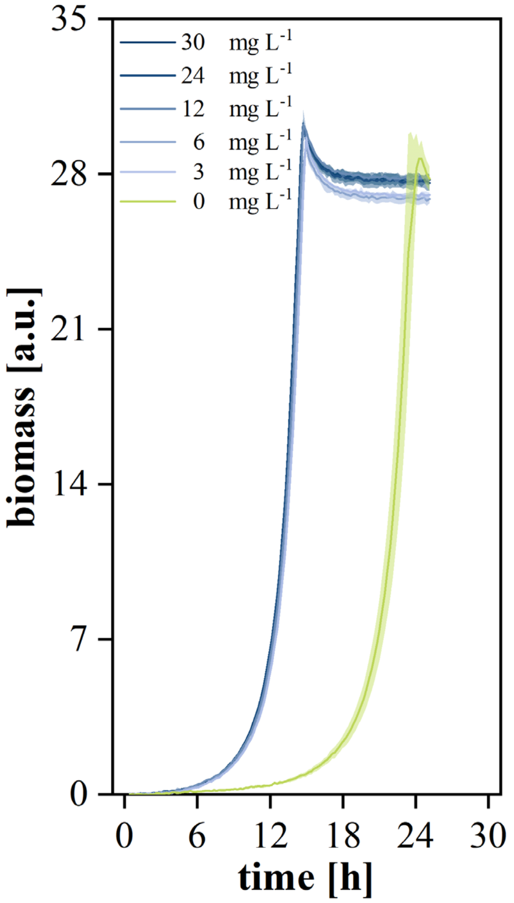

Supplementary figure S2: Batch growth of non-induced *Corynebacterium glutamicum* at varying PCA concentrations; 20 g L^{-1} glucose, 30 °C, 1300 rpm, V_L = 800 µL, relative humidity ≥ 85 %. Error bars deviated from biological replicates (n ≥ 4).
